# Commentary: The apolipoprotein A-I mimetic peptide, D-4F, restrains neointimal formation through heme oxygenase-1 up-regulation

**DOI:** 10.3389/fphar.2017.00708

**Published:** 2017-09-29

**Authors:** Giovanni Li Volti, Roberto Avola, Daniele Tibullo

**Affiliations:** Department of Biomedical and Biotechnological Sciences, University of Catania, Catania, Italy

**Keywords:** endothelium, vascular, smooth muscle cells, neointima formation, restenosis, vascular diseases

We read with great interest the work of Liu et al. (2017b) showing that D-4F inhibited vascular smooth muscle cells (VSMC) proliferation and migration *in vitro* and neointimal formation *in vivo* through heme oxygenase-1(HO-1) up-regulation. Authors' conclusions further demonstrate that HO-1 represents a druggable target for vascular injury prevention and that D-4F may be exploited as a safe and effective treatment to induce HO-1 into a clinical setting. In fact, previous reports showed that a single dose of D-4F is safe and well tolerated in patients with coronary heart disease (Bloedon et al., 2008; Sherman et al., 2010). Furthermore, D-4F, besides being an inducer of HO-1, exhibited pleiotropic effects contributing to vascular homeostasis such as antioxidant and anti-inflammatory effects (Kruger et al., 2005; Rosenbaum et al., 2015; Liu et al., 2017a). Interestingly, all these effects are consistent with HO-1 biological functions. In particular, HO-1 may be expressed under basal conditions (Maines et al., 1986; Bauer et al., 1998) and it is induced by different compounds and stress-related stimuli (Li Volti et al., 2008; Bramanti et al., 2012; Barbagallo et al., 2013). In addition, a number of natural antioxidant compounds contained in foods and plants have been demonstrated to be effective non-stressful and non-cytotoxic inducers of the response protein HO-1 in various cellular models. Most of these compounds are contained in plants, which besides having been widely used as food, spices, or flavoring also represent locally traditional medicinal plants. However, such compounds have intrinsic antioxidant and anti-inflammatory effects because of their ability to induce antioxidant responsive elements (ARE) which are responsible for cellular homeostasis maintenance; on the other hand, D-4F has specific activity on HO-1 induction. Finally, one more aspects deserves to be pointed out referring to the interesting work of Liu et al. Noteworthy, the biological effects of HO-1 have been shown to be cell specific. In fact, several lines of evidence are consistent with the authors' conclusion regarding the effect of HO-1 on VSMC proliferation and migration *in vitro* (Li Volti et al., 2002; Durante, 2010). On the other hand, HO-1 induction results in a significant increase of endothelial cell proliferation (Li Volti et al., 2002, 2005). Such particular cell specificity is of great clinical interest in the case of vascular injury and neointima formation since this process is characterized by increased VSMC proliferation with a reduction of endothelial formation. To this regard, Duckers et al. (2001) showed that HO-1 reduced the proliferative response to vascular injury *in vivo* and reduced VSMC proliferation *in vivo* via p21 regulation. Taken all together, the work of Liu et al. provide a significant pharmacological tool to exploit HO-1 beneficial effects into a clinical setting with particular regard to restenosis.

## Author contributions

GL, RA, and DT reviewed scientific literature and contributed to the writing of this article.

### Conflict of interest statement

The authors declare that the research was conducted in the absence of any commercial or financial relationships that could be construed as a potential conflict of interest.

## References

[B1] Barbagallo I. Galvano F. Frigiola A. Cappello F. Riccioni G. Murabito P.. (2013). Potential therapeutic effects of natural heme oxygenase-1 inducers in cardiovascular diseases. Antioxid. Redox Signal. 18, 507–521. 10.1089/ars.2011.4360 23025298

[B2] Bauer I. Wanner G. A. Rensing H. Alte C. Miescher E. A. Wolf B.. (1998). Expression pattern of heme oxygenase isoenzymes 1 and 2 in normal and stress-exposed rat liver. Hepatology 27, 829–838. 10.1002/hep.510270327 9500714

[B3] Bloedon L. T. Dunbar R. Duffy D. Pinell-Salles P. Norris R. DeGroot B. J.. (2008). Safety, pharmacokinetics, and pharmacodynamics of oral apoA-I mimetic peptide D-4F in high-risk cardiovascular patients. J. Lipid Res. 49, 1344–1352. 10.1194/jlr.P800003-JLR200 18323573 PMC2386905

[B4] Bramanti V. Tomassoni D. Grasso S. Bronzi D. Napoli M. Campisi A.. (2012). Cholinergic precursors modulate the expression of heme oxigenase-1, p21 during astroglial cell proliferation and differentiation in culture. Neurochem. Res. 37, 2795–2804. 10.1007/s11064-012-0873-3 22956150

[B5] Duckers H. J. Boehm M. True A. L. Yet S. F. San H. Park J. L.. (2001). Heme oxygenase-1 protects against vascular constriction and proliferation. Nat. Med. 7, 693–698. 10.1038/89068 11385506

[B6] Durante W. (2010). Targeting heme oxygenase-1 in vascular disease. Curr. Drug Targets 11, 1504–1516. 10.2174/1389450111009011504 20704550 PMC2978667

[B7] Kruger A. L. Peterson S. Turkseven S. Kaminski P. M. Zhang F. F. Quan S.. (2005). D-4F induces heme oxygenase-1 and extracellular superoxide dismutase, decreases endothelial cell sloughing, and improves vascular reactivity in rat model of diabetes. Circulation 111, 3126–3134. 10.1161/CIRCULATIONAHA.104.517102 15939814

[B8] Liu D. Ding Z. Wu M. Xu W. Qian M. Du Q.. (2017a). The apolipoprotein A-I mimetic peptide, D-4F, alleviates ox-LDL-induced oxidative stress and promotes endothelial repair through the eNOS/HO-1 pathway. J. Mol. Cell. Cardiol. 105, 77–88. 10.1016/j.yjmcc.2017.01.017 28274624

[B9] Liu D. Wu M. Du Q. Ding Z. Qian M. Tong Z.. (2017b). The apolipoprotein A-I mimetic peptide, D-4F, restrains neointimal formation through heme oxygenase-1 up-regulation. J. Cell. Mol. Med. [Epub ahead of print]. 10.1111/jcmm.13290 28767201 PMC5706511

[B10] Li Volti G. Sacerdoti D. Di Giacomo C. Barcellona M. L. Scacco A. Murabito P.. (2008). Natural heme oxygenase-1 inducers in hepatobiliary function. World J. Gastroenterol. 14, 6122–6132. 10.3748/wjg.14.6122 18985801 PMC2761572

[B11] Li Volti G. Sacerdoti D. Sangras B. Vanella A. Mezentsev A. Scapagnini G.. (2005). Carbon monoxide signaling in promoting angiogenesis in human microvessel endothelial cells. Antioxid. Redox Signal. 7, 704–710. 10.1089/ars.2005.7.704 15890016

[B12] Li Volti G. Wang J. Traganos F. Kappas A. Abraham N. G. (2002). Differential effect of heme oxygenase-1 in endothelial and smooth muscle cell cycle progression. Biochem. Biophys. Res. Commun. 296, 1077–1082. 10.1016/S0006-291X(02)02054-5 12207883

[B13] Maines M. D. Trakshel G. M. Kutty R. K. (1986). Characterization of two constitutive forms of rat liver microsomal heme oxygenase. Only one molecular species of the enzyme is inducible. J. Biol. Chem. 261, 411–419. 3079757

[B14] Rosenbaum M. A. Chaudhuri P. Abelson B. Cross B. N. Graham L. M. (2015). Apolipoprotein A-I mimetic peptide reverses impaired arterial healing after injury by reducing oxidative stress. Atherosclerosis 241, 709–715. 10.1016/j.atherosclerosis.2015.06.018 26125413 PMC4529116

[B15] Sherman C. B. Peterson S. J. Frishman W. H. (2010). Apolipoprotein A-I mimetic peptides: a potential new therapy for the prevention of atherosclerosis. Cardiol. Rev. 18, 141–147. 10.1097/CRD.0b013e3181c4b508 20395699

